# Prediction of multiple types of drug interactions based on multi-scale fusion and dual-view fusion

**DOI:** 10.3389/fphar.2024.1354540

**Published:** 2024-02-16

**Authors:** Dawei Pan, Ping Lu, Yunbing Wu, Liping Kang, Fengxin Huang, Kaibiao Lin, Fan Yang

**Affiliations:** ^1^ School of Computer and Information Engineering, Xiamen University of Technology, Xiamen, China; ^2^ School of Economics and Management, Xiamen University of Technology, Xiamen, China; ^3^ College of Computer and Big Data, Fuzhou University, Fuzhou, China; ^4^ Pasteur Institute, Soochow University, Suzhou, China; ^5^ Shenzhen Research Institute of Xiamen University, Shenzhen, China; ^6^ Department of Automation, Xiamen University, Xiamen, China

**Keywords:** drug drug interaction prediction, graph neural network, multi-scale fusion, graph features represent learning, multi-class classification

## Abstract

Potential drug-drug interactions (DDI) can lead to adverse drug reactions (ADR), and DDI prediction can help pharmacy researchers detect harmful DDI early. However, existing DDI prediction methods fall short in fully capturing drug information. They typically employ a single-view input, focusing solely on drug features or drug networks. Moreover, they rely exclusively on the final model layer for predictions, overlooking the nuanced information present across various network layers. To address these limitations, we propose a multi-scale dual-view fusion (MSDF) method for DDI prediction. More specifically, MSDF first constructs two views, topological and feature views of drugs, as model inputs. Then a graph convolutional neural network is used to extract the feature representations from each view. On top of that, a multi-scale fusion module integrates information across different graph convolutional layers to create comprehensive drug embeddings. The embeddings from the two views are summed as the final representation for classification. Experiments on two real-world datasets demonstrate that MSDF achieves higher accuracy than state-of-the-art methods, as the dual-view, multi-scale approach better captures drug characteristics.

## 1 Introduction

The concurrent use of multiple medications is common, as combining drugs can reduce individual dosages and toxicity (Chou and Talalay, 1983). However, drug-drug interactions can alter potency and lead to adverse reactions (Lazarou et al., 1998). Moreover, as polypharmacy rises, so does the likelihood of adverse drug-drug interactions. For example, in the United States, approximately 74,000 emergency visits and 195,000 hospitalizations annually stem from antagonistic DDIs (Percha and Altman, 2013). Consequently, accurate identification of DDI is critical. Nevertheless, traditional *in vitro* and clinical diagnosis methods for DDI detection are often expensive and time-consuming (Huang et al., 2020b).

In recent years, deep learning has shown strong performance on prediction tasks (Dong et al., 2022; Meng et al., 2022). The deep learning technique, as one of the most advanced computational methods, has demonstrated a good performance on prediction tasks (Deng et al., 2020), bringing new solutions to the DDI prediction. When applying deep learning for DDI prediction, three main information types are now used: i) drug features like chemical substructures, enzymes, and targets (Lin S. et al., 2022b); ii) knowledge graphs containing DDI information (Wang et al., 2023); iii) DDI networks (Xiong et al., 2023).

While the above-mentioned methods have yielded good results, there are still two unresolved deficiencies. First, most graph neural network-based approaches(Feng et al., 2020) rely solely on the DDI graphs as inputs, which limits their ability to capture the full spectrum of drug interactions. This is because some DDIs may not yet be identified, leading to incomplete topological features. Second, current methods do not leverage multi-scale information that emerges during the process of information propagation. In graph neural networks, feature vectors from different network layers vary in dimensionality, semantics, and other informational scales. This variability is known as multi-scale information (Peng et al., 2021). Shallow network layers yield feature vectors with low-scale, more basic information, while deeper layers provide high-scale, more semantically rich information. By integrating information from these various scales, a more comprehensive understanding can be achieved. However, existing DDI prediction models predominantly utilize feature vectors from the final network layer, which only represent high-scale information. Although this information is less complex and rich in semantic features, it overlooks the detailed insights offered by low-scale information.

To solve the above problems, this paper introduces a new DDI prediction model, named MSDF, which employs multi-scale and two-view fusion techniques. First, alongside the DDI graph, we construct a feature similarity graph to supplement topological information. We base our approach on the assumption that drug pairs likely to interact exhibit high feature similarity. By connecting these similar nodes, the feature graph includes potential, yet undiscovered DDIs, thus addressing the issue of incomplete topological data in a standalone DDI graph. However, it is important to note that not all drugs with high feature similarity will interact. Hence, during the construction of the feature graph, some non-interacting drug pairs may be erroneously linked. This issue is mitigated by combining the feature graph with the more accurate topological data from the DDI graph, allowing for a correction of any inaccuracies in the feature graph. Overall, the complementary views improve predictions when fused. Second, we extract and utilize multi-scale insights across network layers from both views, which differ in dimensionality and semantics. Attention mechanisms then fuse these multi-scale representations to create comprehensive drug embeddings. In this way, the drug node representations integrate both localized and high-level insights for comprehensive learning, and are more favorable for the final DDI prediction task. Our contributions are summarized as follows.

(1) We propose a new DDI prediction model: the MSDF. The model introduces a multi-scale fusion module, so that the node representations of the drug can contain structural and semantic information at different scales.
(2) MSDF fuses multi-scale information from two views (DDI topology and feature views), and experimental results show that this approach facilitates model performance.
(3) The model in this paper accomplishes the prediction of binary classification and multi-classification on both DeepDDI and DDIMDL datasets, and achieves better performance than the baseline method on both tasks, reflecting the sophistication and comprehensiveness of the model.

The remainder of the paper is organized as follows. Section 2 reviews related work in DDI prediction. Section 3 details the methodology behind MSDF. Section 4 presents the conducted experiments. Section 5 provides a conclusion and outlines the future directions.

## 2 Related work

Existing deep learning-based DDI prediction methods can be basically categorized into three types: methods based on drug feature information, methods that fuse knowledge graph, and methods based on topological features.

The drug feature similarity approach assumes that drugs with potential interactions share similar characteristics. This method employs deep learning techniques to distill drug features for classification tasks. Commonly used drug features include drug category (Zhang et al., 2022), chemical structure (Vilar et al., 2012), side effects (Tatonetti et al., 2011), and profile fingerprints (Vilar et al., 2013). Initially, these methods often focused on a single feature. For example, Ryu et al. (Ryu et al., 2018) developed the DeepDDI model, which uses the chemical substructures of drug pairs to calculate structural similarity profiles (SSPs). These SSPs are then fed into a Deep Neural Network (DNN) for predicting interactions. More recent research has highlighted the benefits of integrating multiple feature sources for improved prediction accuracy (Gottlieb et al., 2012; Cheng and Zhao, 2014; Yan et al., 2020). For instance, Lee et al. (Lee et al., 2019) combined SSPs, target genes, and gene ontology, encoding each feature separately using an AutoEncoder (AE) for classification. Deng et al. (Deng et al., 2020) utilized a dataset from Drugbank (Wishart et al., 2018) with 37,264 DDI events. They employed the StandfordNLP (Qi et al., 2018) tool to categorize these events into 65 types, using this classification as labels for model predictions. The model incorporated features like chemical substructures, enzymes, and drug targets, processed through a DNN. Furthermore, Yang et al. (Yang et al., 2023) built upon Deng et al.’s (Deng et al., 2020) dataset, employing Convolutional Neural Networks (CNNs) to extract drug feature vectors, resulting in enhanced prediction outcomes.

Methods based on drug feature similarity often overlook the crucial topological information represented as the DDI graph, which indicates the likelihood of interactions between drugs. However, incorporating this topological data can significantly enhance the accuracy of DDI predictions. Additionally, the choice and combination of drug features profoundly affect the model’s performance. More specifically, Deng et al. (Deng et al., 2020) observed that models using a diverse range of features tend to yield better results than those relying on a single feature. Nevertheless, it’s also important to note that simply adding more features does not always lead to improved outcomes; in some cases, it may even diminish the model’s effectiveness. Thus, careful selection and integration of multi-source features are essential. This process requires a deep understanding and extensive experience in the field, as the model designer must balance the quantity and quality of features to optimize the model’s performance.

In the method of fusion knowledge graph, a knowledge graph is first constructed from the dataset. This knowledge graph includes not only drug entities but also various heterogeneous nodes, such as target and transporter nodes. By extracting information from the knowledge graph, a drug node can assimilate a richer array of information from these diverse nodes. Once the knowledge graph is established, it serves as an input for predictive models. The knowledge graph fusion method typically employs traditional knowledge graph embedding techniques, such as TransE (Bordes et al., 2013), ComplEx (Trouillon et al., 2016), RotatE (Sun et al., 2018) or utilize graph neural network approaches to learn the feature vectors of drug nodes within the graph. For instance, Yu et al. (Yu et al., 2022) developed the RANEDDI model, which uses the RotatE method for initializing drug embeddings in the knowledge graph and then feeds these embeddings into a relation-aware network for DDI prediction. Similarly, Hong et al. (Hong et al., 2022) introduced LaGAT, a graph attention model that employs the knowledge graph as its input. LaGAT generates various attention paths for drug entities by aggregating information from neighboring nodes, tailored to different drug pairs. Yao et al. (Yao J. et al., 2022) took a different approach by modeling drug pairs and their side effects within a knowledge graph, using nonlinear functions for semantic transfer, thereby demonstrating effective scalability in extensive knowledge graphs. While the knowledge graph fusion method successfully integrates a broader spectrum of heterogeneous information, it also introduces certain challenges. These include the potential for increased noise in the data and the complexity involved in constructing and managing the knowledge graph.

Drug topology feature-based approaches (Zitnik et al., 2018; Yue et al., 2020) usually use graph embedding techniques [e.g., Deepwalk (Perozzi et al., 2014), Node2Vec (Grover and Leskovec, 2016), SDNE (Wang et al., 2016), GCN (Kipf and Welling, 2023), GAT (Veličković et al., 2017), etc.] to extract topological features of drug nodes in DDI network for DDI prediction. Earlier studies, often preferred traditional graph embedding methods to extract topological features of drugs. For example, Park et al. (Park et al., 2015) used a random wandering algorithm and a restart algorithm to extract topological features of drugs on protein-protein interaction networks. However, these topological feature-based methods typically only depend on the topological feature. In other words, they tend to overlook the drug’s own attributes, such as enzymes and targets, leading to incomplete analysis. Consequently, attribute graph-based methods have gained popularity. The attribute graph-based methods enhance a topology graph by adding node attributes, thereby incorporating the drug’s own attributes into the analysis. This integration of additional information makes attribute graphs more effective for DDI prediction than traditional topological graphs. Graph neural network approaches, as opposed to traditional graph embedding methods like Deepwalk or Node2Vec, are better suited to handle attribute graphs. They can process both the topology and node attributes simultaneously, extracting comprehensive drug node information through multi-layer aggregation and update operations. Recently, the trend has shifted towards combining graph neural networks with attribute graphs. A notable example is the work of Wang et al. (Wang et al., 2022) who created DDI increasing and decreasing graphs based on DDI types and employed GCN to learn drug representations using drug targets as node features. However, challenges persist in this evolving field. Wang et al. (Wang et al., 2020) noted that GCN’s fusion mechanism sometimes fails to effectively integrate node features and topological information. Furthermore Yao et al. (Yao K. et al., 2022) pointed out that topological graphs might suffer from incomplete information, leading to suboptimal embeddings of learned nodes and negatively impacting downstream applications.

Therefore, in order to address the problems mentioned above, our model incorporates both a topological graph and a feature graph as inputs. By introducing a feature graph, the feature matrices of the nodes are propagated in both graphs simultaneously and the information from both graphs is complemented in order to improve the performance of the model. Moreover, in contrast to existing graph neural network-based models, which typically use only the output of the last network layer for classification tasks, we develop a multi-scale information fusion module. This module is designed to merge information from different scales, thereby enabling more accurate predictions.

## 3 Materials and methods

### 3.1 Overview

**FIGURE 1:**
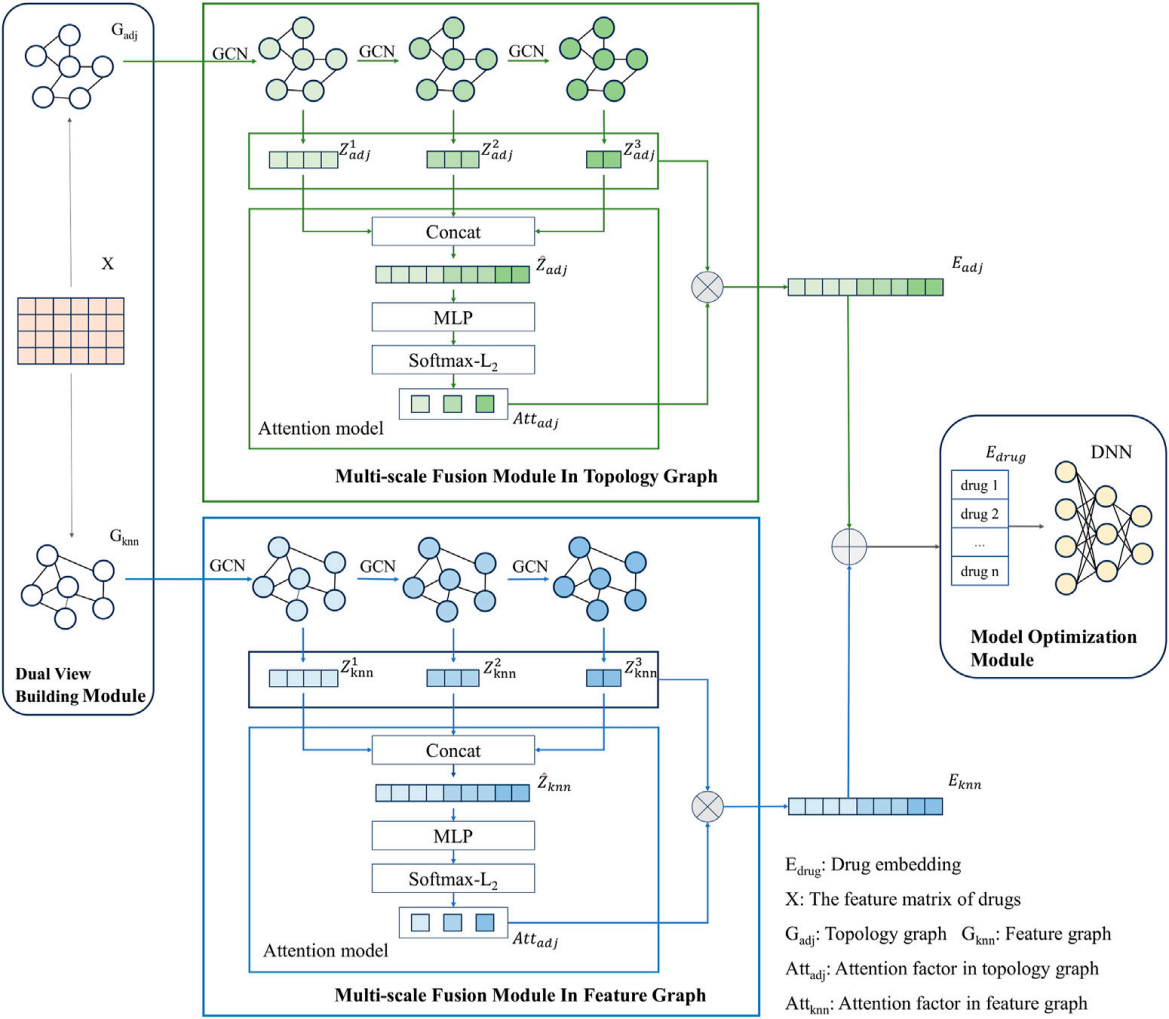
Model diagram of MSDF.

The modeling framework of MSDF is shown in Figure 1. It consists of three modules: dual view construction module, multi-scale fusion module, and model optimization module. The process begins with the dual view construction module, where two views of the drug information are generated: the topological graph ( $G_{\text{adj}}$ ) and the feature graph ( $G_{\text{knn}}$ ). These graphs, along with the drug feature matrix, are then fed into the multi-scale fusion module. In the multi-scale fusion module, the graphs and drug feature matrices undergo a series of operations including feature aggregation, updating, and cycling. These operations are performed using a GCN to derive multi-scale information from the drug nodes. To enhance the integration of information across different scales, the model incorporates an attention mechanism. This mechanism assigns weights, or “attention factors,” to different scales based on their contribution to the final outcome. Each feature vector at a specific scale is then multiplied by its corresponding attention factor, creating a scaled vector representation. Subsequently, these scaled vector representations from different scales are concatenated using a splice operation. This results in a comprehensive final node representation for each drug node in that particular view. Once the multi-scale representations for both views are obtained, they are combined through a summation operation to form the final vector representations of the drug nodes. Finally, for the DDI classification task, the feature vectors of drug pairs are merged and inputted into a DNN. This is done based on the labeling information provided in the dataset, completing the classification process.

### 3.2 Problem formulation

In this section, we define the problem using the notations provided in Table 1. We consider to a DDI network represented as $G=\left\{V,E,X\right\}$ . Here, V denotes the drug nodes in the DDI network, E denotes the edges between the drug nodes, and X is the feature matrix associated with the drugs. The objective of the MDSF accurately predict DDIs through both binary and multi-classification approaches. In more detail, the aim of binary classification is to determine whether a connection exists between any two given drug nodes, i.e., whether a DDI will occur. For multi classification, the goal is to identify multi-classification is to the specific type of DDI that will occur between a pair of interacting drugs.

**TABLE 1:** Key symbols and definitions.

Notation	Definition and description
G	The input network
V	The node set
E	The edge set
X	Attribute feature of nodes
$s_{\text{ij}}$	The feature similarity of drug i, j vectors
$Z_{y}^{\left(l\right)}$	The embedding learned by GCN in view y at layer l
${A\text{tt}}_{y}$	Attention factor for different scales of information in view y
$E_{y}$	Embedding of nodes under y-view
$E_{\text{drug}}$	The unified embedding

### 3.3 Data pre-processing

The features of a drug can be represented by a set of descriptors. In this representation, when a certain feature is present in a drug, the description of the drug in this feature is set to 1, and *vice versa* to 0. For instance, consider the drug Etodolac in the DDIMDL dataset, which lists 1162 potential targets. Etodolac is associated with three specific targets: P35354, P23219, and P19793. In its feature vector, the positions corresponding to these three targets are set to “1,” while the rest of the positions, representing the other 1159 targets, are set to “0.” While this descriptor-based approach accurately captures the features of a drug, it has a notable drawback: the resulting feature vectors are high-dimensional and predominantly filled with zeros. This leads to the “curse of dimensionality,” a phenomenon where the high number of dimensions (features) negatively impacts the model’s performance due to the sparsity of data. To mitigate this issue, it is essential to preprocess the drug features, aiming to reduce the dimensionality of the feature vectors.

The method of pre-processing in this paper is to compute the Jaccard similarity of drug features and then use the resulting Jaccard similarity matrix as the node features for each drug. This approach is applied to two datasets: the DDIMDL dataset extracted by Deng et al. (Deng et al., 2020) and the DeepDDI dataset extracted by Ryu et al. (Ryu et al., 2018) Detailed information about the datasets will be presented in the experimental section. For the DDIMDL dataset (Deng et al., 2020), this paper calculates the Jaccard similarity matrices of drug chemical substructures, enzymes, and targets, respectively, and splices the Jaccard similarity matrices of the three features as the feature vectors of the drugs in this dataset. For DeepDDI dataset (Ryu et al., 2018), since this dataset has only one feature of chemical substructures, this paper calculates the Jaccard similarity matrix of this feature as the feature vector of the drug in this dataset. The formula for calculating the Jaccard similarity is shown in Eq. 1. In Eq. 1, $d_{i}$ and $d_{j}$ are the initial feature vectors of drug i and drug j, ∩ is the intersection operation, and ∪ is the concatenation operation.

$$J\left(d_{i},d_{j}\right)=\frac{\left|d_{i}\cap d_{j}\right|}{\left|d_{i}\cup d_{j}\right|}=\frac{\left|d_{i}\cap d_{j}\right|}{\left|d_{i}\right|+\left|d_{j}\right|-\left|d_{i}\cap d_{j}\right|}$$ (1)

### 3.4 Dual view building module

In order to represent the topological relationships between DDIs and the similarity of features between drugs, this paper constructs a topological graph of DDIs as well as a feature graph between drugs. These graphs are then utilized as inputs to the model for subsequent prediction tasks. The construction of the two views is described below.

#### 3.4.1 Topology graph construction

The topology graph represents a kind of localized structural information between DDIs, denoted as $G_{\text{adj}}$ . It is constructed by creating an adjacency matrix whose rows and columns are represented as drug nodes. In this matrix, an entry is set to “1” if there is a DDI between the corresponding drugs in a row and a column, indicating a connection. Conversely, an entry is marked as “0” if there is no interaction. Thus, the adjacency matrix can be represented as the topology graph of the DDI.

#### 3.4.2 Feature graph construction

The feature graph represents the similarity of drug nodes in the feature space, denoted as $G_{\text{knn}}$ . It is constructed by traversing all the drug nodes. During this process, we compute the feature similarity between each traversed drug node and all other nodes in the dataset. Upon completing this traversal, we obtain a similarity score for each pair of drug nodes. To construct the feature graph, also known as a K-Nearest Neighbors (KNN) graph, we connect each drug node to its top-K most similar nodes, forming edges based on these high similarity scores. Note that, the method used for calculating the similarity between drug nodes is the cosine similarity, as shown in Eq. 2.

$$s_{ij}=\frac{X_{i}∙X_{j}}{\left\|X_{i}\right\|\left\|X_{j}\right\|}$$ (2)

Where $s_{\text{ij}}$ is the feature similarity score of drug i and drug j, $X_{i}$ is the feature vector of drug i, $X_{j}$ is the feature vector of drug j, and the symbol   denotes the Euclidean paradigm operation.

### 3.5 Multi-scale fusion module

After obtaining the feature matrix of the drug and the two graphical views - the topological graph and the feature graph -, our model proceeds to extract and fuse these features for the final DDI prediction task. To facilitate this, we have designed a multi-scale fusion module.

First, we use GCN to extract the drug node information in the network. For the topological graph, the output of GCN at each layer of the network is denoted as $Z_{\text{adj}}^{\left(l\right)}$ , where l represents the layer number. Taking the topological graph as an example, the formulation of GCN can be represented by Eqs 3, 4.

$$\tilde{A}=D^{-\frac{1}{2}}\left(A+I\right)D^{-\frac{1}{2}}$$ (3)

$$Z_{adj}^{\left(l\right)}=\sigma \left(\tilde{A}Z_{adj}^{\left(l-1\right)}W^{\left(l\right)}\right)$$ (4)

In Eq. 3, A denotes the adjacency matrix, I is the diagonal matrix of A , and D is the degree matrix of A . In Eq. 4, $Z_{\text{adj}}^{\left(l\right)}$ denotes the output of the l _th_ layer in the GCN, $Z_{\text{adj}}^{\left(l-1\right)}$ denotes the output of the $\left(l-1\right)$ _th_ layer in the GCN, $Z_{\text{adj}}^{\left(0\right)}$ is the node feature matrix X , $W^{\left(l\right)}$ denotes the learnable parameter matrix in the GCN, and σ is the activation function.

**FIGURE 2:**
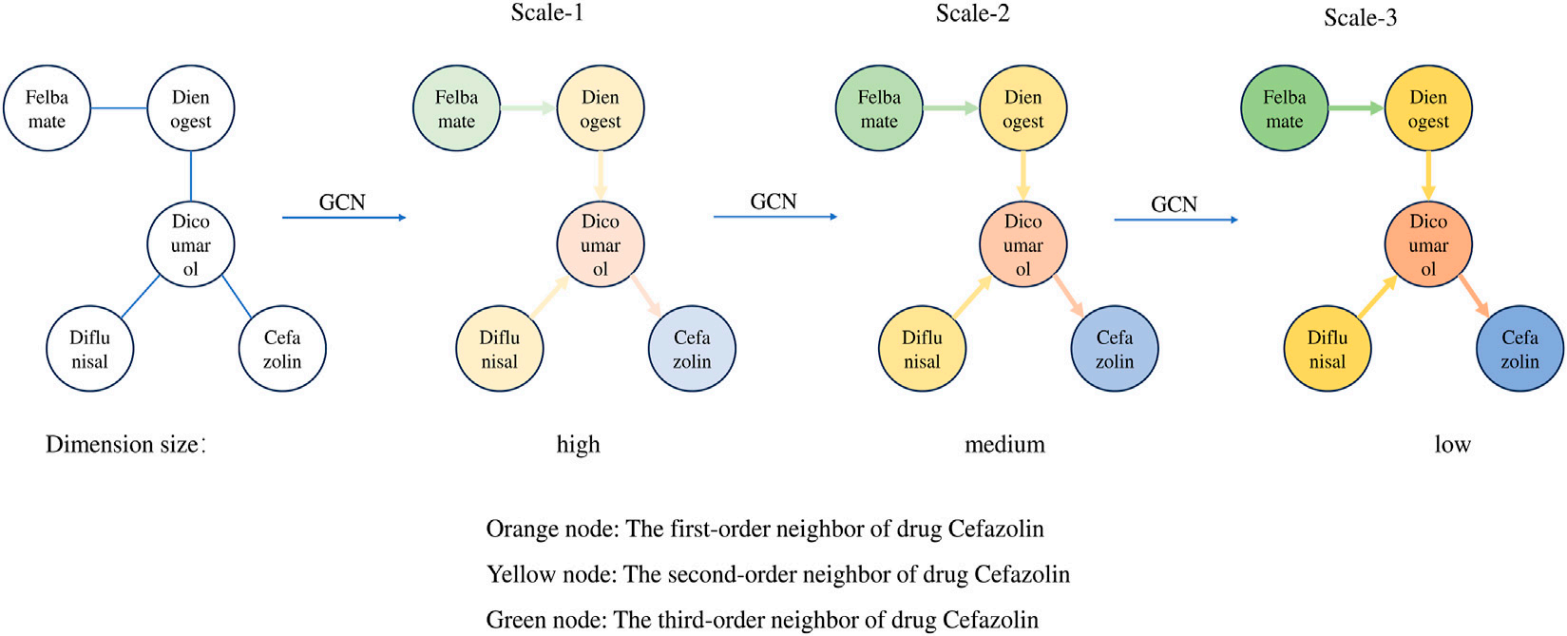
Aggregation process and scale information of target node Cefazolin.

To illustrate the information aggregation process in GCNs, this paper selects five drugs from Drugbank to construct a DDI subgraph. In this subgraph, Cefazolin is treated as the target node, with its neighboring nodes of different orders represented by varying colors. This approach allows us to visually examine how target nodes in GCNs aggregate information from neighboring nodes at different layers. As can be seen from Figure 2, with a single GCN layer, the target node Cefazolin only aggregates information from its first-order neighbor Dicoumarol. However, as we increase the number of GCN layers, Cefazolin begins to incorporate information from higher-order neighbors. For instance, at two GCN layers, Cefazolin continues to aggregate information from its first-order neighbor Dicoumarol. But since Dicoumarol has already aggregated information from its own first-order neighbors (Dienogest and Diflunisal) in the first GCN layer, Cefazolin indirectly aggregates second-order neighbor information via the graph convolution in the second layer. Additionally, GCN serves a role in reducing the dimensionality of feature vectors. As the number of GCN layers increases, the feature vector dimensionality of the target node Cefazolin is progressively reduced from high to low. Therefore, we observe two key changes in node characteristics within the DDI network as the number of GCN layers increases:

• The target node obtains information about its higher-order neighbors as the number of GCN layers increases;
• The feature dimension of the target node decreases as the number of GCN layers increases.

With the two changes summarized above, it is known that as the number of GCN layers increases, the network generates information with different characteristics at different scales. In more detail, higher scales will contain information about higher-order neighbors. This results in feature vectors with lower dimensionality but richer in semantic content, although they may lose some detailed information. In contrast, at lower scales, the information is limited to lower-order neighbors, retaining more detailed data in the feature vectors due to their higher dimensionality, albeit with a slight increase in noise. Intuitively, feature vectors at higher scales are of superior quality as they encompass a broader scope of neighboring node information. However, unlike in other deep learning networks like CNNs, where the network depth can extend to dozens or even hundreds of layers, a GCN typically does not exceed 5 layers. The reason is the occurrence of “over-smoothing” as the number of layers increases. In higher GCN layers, the node representations tend to become overly similar, diminishing their ability to effectively perform subsequent downstream tasks. This phenomenon and its impact on node feature vectors are further corroborated by the experiments conducted in this paper.

As mentioned above, different scales of information in the GCN network have their own advantages and shortcomings for the final prediction results. By integrating information from various scales, we can maximize the benefits of each scale while compensating for their respective shortcomings. By doing so, our approach marks a departure from traditional practices in graph neural networks, where only the output from the network’s last layer is typically used as the final node vector. In contrast, our paper emphasizes the utility of outputs from each layer of the GCN, processing them collectively to enhance the accuracy of subsequent DDI predictions.

While multi-scale information contributes to the final prediction in our model, it is important to note that information from different scales has varying degrees of impact on the results. Drawing inspiration from Peng et al. (Peng et al., 2021), our paper incorporates an attention mechanism to more effectively integrate information across multiple scales. First of all, due to the varied dimensions of information at different scales, a direct approach to unifying this multi-scale information is not feasible. Instead, we concatenate the feature vectors from different scales to create a unified input for the attention mechanism module. To be more specific, taking the topological graph as an example, we splice the feature vectors from different scales as demonstrated in Eq. 5.

$${\hat{Z}}_{adj}=\left[\left.Z_{adj}^{1}\right\|\left.Z_{adj}^{2}\right\|∙∙∙\left\|Z_{adj}^{l}\right.\right]$$ (5)

In Eq. 5, $Z_{\text{adj}}^{l}$ denotes the output of the l _th_ layer of the GCN in the topological graph, ∥ denotes the splicing operation, and ${\hat{Z}}_{\text{adj}}$ is the final vector after splicing of the multiscale feature vectors. After obtaining ${\hat{Z}}_{\text{adj}}$ , we input ${\hat{Z}}_{\text{adj}}$ into the fully-connected layer, the dimension of the fully-connected output layer is the number of layers l of the GCN. The output is the attention factor of the output of each layer of the GCN. After obtaining the attention factors, this paper uses the softmax function to perform the normalization operation and then obtains the final attention factors by $l_{2}$ regularization, which is calculated via Eq. 6.

$${Att}_{adj}=l_{2}\left(softmax\left(LeakyRelu\left(linear\left({\hat{Z}}_{adj}\right)\right)\right)\right)$$ (6)

In Eq. 6, ${A\text{tt}}_{\text{adj}}$ represents the matrix of attention factors in the topological graph, which can be expressed as $\left[\left.a_{\text{adj}}^{1}\right\|\left.a_{\text{adj}}^{2}\right\|∙∙∙\left\|a_{\text{adj}}^{l}\right.\right]$ , and the data in each column of ${A\text{tt}}_{\text{adj}}$ represent the attention factors of the eigenvectors of the corresponding scales. By the same method, we can get the attention factor matrix in the feature graph as ${A\text{tt}}_{\text{knn}}$ . In this paper, the attention factors are multiplied with the corresponding scale information and spliced into the final embedding form, the embedding vector $E_{\text{adj}}$ in the topology graph and the embedding vector $E_{\text{knn}}$ in the feature graph can be expressed by Eqs 7, 8.

$$E_{adj}=a_{adj}^{1}∙Z_{adj}^{1}\left\|a_{adj}^{2}∙Z_{adj}^{2}\left\|∙∙∙\right.\right.\left\|a_{adj}^{l}∙Z_{adj}^{l}\right.$$ (7)

$$E_{knn}=a_{knn}^{1}∙Z_{knn}^{1}\left\|a_{knn}^{2}∙Z_{knn}^{2}\left\|∙∙∙\right.\right.\left\|a_{knn}^{l}∙Z_{knn}^{l}\right.$$ (8)

After obtaining the embedding vector $E_{\text{adj}}$ of the drug for the topological graph and the embedding vector $E_{\text{knn}}$ for the feature graph, we use summation to realize the fusion of the embedding vectors learned by the model in the two views, as shown in Eq. 9.

$$E_{drug}=E_{adj}+E_{knn}$$ (9)

### 3.6 Model optimization module

Once obtaining the final embedding vector $E_{\text{drug}}$ for each drug, our next step is to predict the DDI based on the labeling information in the dataset. To do this, we first combine the embeddings of two drugs that are likely to interact. This combination is achieved using a specific function, as outlined in Table 2. We express the combined drug pairs as shown in Eq. 10, where $\text{aggregation}\left(\right)$ represents the combination function, $E_{\text{drug}\;i}$ is the embedding vector of drug i, $E_{\text{drug}\;j}$ is the embedding vector of drug j, and the resultant drug pairs are denoted as DPs. For the binary classification task, the activation function of the last layer of the DNN is the sigmoid function, and its prediction can be expressed as Eq. 11. For the multi-classification task, the activation function of the last layer of the DNN is a softmax function, and its prediction result can be expressed as Eq. 12.

$$DPs=aggregation\left(E_{drug\;i},E_{drug\;j}\right)$$ (10)

$${\hat{y}}_{ij}^{b}=sigmoid\;\left(DNN\left(DPs\right)\right)$$ (11)

$${\hat{y}}_{ij}^{m}=softmax\left(DNN\left(DPs\right)\right)$$ (12)

**TABLE 2:** The way the three drug pairs were combined.

Combination method	Dimensionality	Description
Concatenation	2d	$\Phi \left(d_{i},d_{j}\right)=\Phi \left(d_{i}\right)\left\|\Phi \left(d_{j}\right)\right.$
Average	d	$\Phi \left(d_{i},d_{j}\right)=0.5\times \left[\Phi \left(d_{i}\right)+\Phi \left(d_{j}\right)\right]$
Hadamard	d	$\Phi \left(d_{i},d_{j}\right)=\Phi \left(d_{i}\right)∙\Phi \left(d_{j}\right)$

In this paper, we address both binary and multi-classification problems, necessitating the use of different loss functions to train the model effectively for each task. For binary classification, we employ the binary cross-entropy loss function. The formula for this function is detailed in Eq. 13. In contrast, for multi-classification tasks, the model uses the cross-entropy loss function, with its formula provided in Eq. 14.

$${Loss}_{b}=-\sum_{\left(d_{i},d_{j}\right)\in \varepsilon }\left(y_{ij}\log\left({\hat{y}}_{ij}\right)+\left(1-y_{ij}\right)\log\left(1-{\hat{y}}_{ij}\right)\right)$$ (13)

$${Loss}_{m}=-\sum_{\left(d_{i},d_{j},r\right)\in \varepsilon }\sum_{c=1}^{R}y_{m}^{c}\log\;\left({\hat{y}}_{m}^{\left(c\right)}\right)$$ (14)

In Eq. 13 $y_{\text{ij}}$ denotes the true label and ${\hat{y}}_{\text{ij}}$ denotes the predicted outcome. In Eq. 14, c is the response type of the drug pair, $y_{m}^{c}$ denotes the true labeling of the drug pair, and ${\hat{y}}_{m}^{\left(c\right)}$ denotes the labeling of the predicted outcome of the model.

## 4 Results and discussion

### 4.1 Datasets

In this paper, experiments were conducted using two datasets of different sizes, and the details of the datasets are shown in Table 3. The larger dataset is the one provided by DeepDDI, in which there are 1710 drugs and 192284 DDI events. These events are categorized into 86 different reaction types, which are used as labels for the models. The drugs in this dataset are characterized by their medicinal chemical substructures. Within the DeepDDI dataset, we conduct experiments for both multi-classification and binary classification tasks. The smaller dataset, obtained from DDIMDL, consists of 572 drugs and 37,264 DDI events, which are classified into 65 reaction types. In the DDIMDL dataset, a total of molecular structures, enzymes, targets, and channels were collected as features, and it was verified that the model achieved the best results when using molecular structures, enzymes, and targets as the drug features according to the experiments of Deng et al. (Deng et al., 2020). Therefore, in this paper, these three features were chosen as the features of the model.

**TABLE 3:** Information on the dataset.

Dataset	Size	Drugs	Interaction	DDI type
DB1	Small	572	37624	65
DB2	Large	1710	192284	86

### 4.2 Baseline methods

In order to verify the effectiveness of the MSDF method proposed in this paper, we have chosen the most advanced DDI prediction algorithms for comparison. The methods chosen are those based on the drug’s own feature: DNN, DeepDDI, and DDIMDL, and attribute graph based methods: SkipGNN, DM-DDI, MDFA, and AM-GCN. Among the methods based on attribute graphs, SkipGNN, MDFA, and AM-GCN are all multi-view methods. Note that, the purpose of introducing multi-view methods is to verify whether the method proposed in this paper is superior under the same multi-view condition. The type information of the baseline method is shown in Table 4, and the details of the baseline methods are shown below:

• DNN: This paper inputs the drug’s own features directly into a DNN to perform both binary and multi-classification tasks for DDI prediction.
• DeepDDI (Ryu et al., 2018): DeepDDI takes the chemical substructures of drugs as features. Moreover, it employs Principal Component Analysis (PCA) to reduce the dimensionality of the feature matrix, which is then input into a DNN for prediction.
• DDIMDL (Deng et al., 2020): As part of a multimodal DDI prediction framework, DDIMDL uses chemical substructures, enzymes, and targets as drug features. It reduces feature sparsity by calculating Jaccard similarity for each feature, followed by inputting these features into a DNN for prediction.
• SkipGNN (Huang et al., 2020a): SkipGNN is a multi-view graph neural network model, which takes into account the existence of jump similarity of nodes in a DDI network. It includes a two-hop neighbor graph in addition to the original topology graph and employs an iterative fusion method for information integration between the two views.
• AM-GCN (Wang et al., 2020): AM-GCN constructs a KNN graph based on node features and designs three channels for extracting information from topological and KNN graphs. The first two channels use GCNs with different parameters for extracting information in topological and KNN graphs. The third channel uses GCNs with shared parameters to extract common information in both graphs and learns the attention factors of the embedding vectors from the three channels using a self-attention mechanism for subsequent downstream tasks.
• DM-DDI (Kang et al., 2022): DM-DDI learns drug node features through AutoEncoder (AE) and topological features through GCN. It inputs outputs from each AE layer into corresponding GCN layers using a deep fusion strategy, which helps mitigate the over-smoothing problem in deep GCNs. The model then uses a self-attention mechanism to fuse topological and node features.
• MFDA (Lin K. et al., 2022a): MFDA constructs three views and uses a cross-fertilization strategy to fuse topological information from the graph and feature information of the drug itself. It introduces dual attention mechanisms at both the node-level and view-level, fusing the topological and feature information of nodes at the node-level, and combining embedding vectors from three views at the view-level for comprehensive drug embeddings.

**TABLE 4:** Information on the type of baseline methods.

Method	Feature type			Task	
	Self-feature	Network	Multi-view	Binary prediction	Multi-class prediction
DNN	√			√	√
DeepDDI	√			√	√
DDIMDL	√				√
SkipGNN	√	√	√	√	
AM-GCN	√	√	√	√	√
DM-DDI	√	√			√
MFDA	√	√	√		√
MSDF	√	√	√	√	√

### 4.3 Assessment of indicators

In order to evaluate the prediction ability of our model, different evaluation metrics are employed for binary and multi-classification tasks in our experiments. For binary classification, the model’s performance is measured using three key indicators: accuracy rate, Area Under the Curve (AUC) of the Receiver Operating Characteristic (ROC) curve, and the Area Under the Precision-Recall Curve (AUCPR).

In multi-classification experiments, a broader set of metrics is utilized, including accuracy, F1 score, precision, recall, AUC curve, and AUCPR curve. Among these metrics, we use macro-averaged measures for F1 score (F1_macro), precision (Pre_macro), and recall (Recall_macro). Conversely, for AUC and AUCPR, we employ micro-averaged metrics, denoted as AUC_micro and AUCPR_micro, respectively. It is noteworthy that in the context of multi-classification tasks, the micro-averaged precision (Pre_micro), recall (Recall_micro), and F1 score (F1_micro) are equivalent to the overall accuracy. Therefore, in our experiments, we opt for macro-averaged metrics for precision, recall, and F1 score to provide a more comprehensive evaluation.

### 4.4 Experimental settings

In this paper, we conducted 5-fold cross-validation, dividing the dataset into 5 equal parts, training on 4 parts and testing on the remaining part. This process was repeated 5 times, with each part used as the test set once. The final model performance was the average across the 5 folds. For the experiments of binary classification, in this paper, unconnected drug pairs are randomly selected as negative samples, and the ratio of positive and negative samples for the experiments is 1:1.

Regarding the model’s parameter settings, we align the DNN parameters with those used in DDIMDL. This includes the incorporation of a batch normalization layer to accelerate convergence and a dropout layer with a rate of 0.3 to prevent overfitting. The activation function used here is the ReLU function. For the final layer, the softmax function is used in multi-classification tasks, while the sigmoid function is applied in binary classification tasks. Specifically for the DDIMDL dataset, the batch size is set to 1000, the epoch is set to 100, and the learning rate is set to 0.001. For the multi-classification task on the DeepDDI dataset, the batch size is set to 512, the epoch is set to 50, and the learning rate is set to 0.001. For the binary classification task on the DeepDDI dataset and the DDIMDL dataset, the batch size is set to 1000, the epoch is set to 50, and the learning rate is set to 0.0001.

### 4.5 Predicted results of DDI binary classification

The experimental results of our model and other baseline models for the task of binary classification are shown in Table 5. It is important to note that the original SkipGNN article, there is did not use the drug’s own features as the node features, and the drug’s node features are set as one-hot encoding in the original article. However, in our experiments, we have employed the drug’s own features as node features in SkipGNN.

**TABLE 5:** Prediction results for binary classification.

Dataset	Method	ACC	AUCPR	AUC
DeepDDI	DNN	0.869	0.932	0.943
	DeepDDI	0.878	0.942	0.946
	SkipGNN	0.947	0.989	0.989
	AM-GCN	0.928	0.982	0.982
	MSDF	**0.959**	**0.993**	**0.993**
DDIMDL	DNN	0.925	0.973	0.974
	DeepDDI	0.864	0.931	0.936
	SkipGNN	0.897	0.971	0.969
	AM-GCN	0.930	0.984	0.982
	MSDF	**0.963**	**0.993**	**0.993**

The best results are shown in bold.

Table 5 demonstrates that in the DeepDDI dataset, methods relying solely on a drug’s own features (DNN and DeepDDI) underperform compared to those utilizing attribute graphs across all metrics. Conversely, in the DDIMDL dataset, DNN’s performance aligns with the attribute graph-based AMGCN, while DeepDDI lags. This discrepancy arises because the DeepDDI dataset benefits from topological information for a holistic drug representation, whereas the DDIMDL dataset is more reliant on intrinsic drug features. Notably, DeepDDI only processes chemical substructures, while DNN, incorporating three drug features, surpasses DeepDDI in the DDIMDL dataset. Across both datasets, our proposed method consistently yields optimal results, substantiating MSDF’s efficacy in binary classification tasks.

### 4.6 Predicted results of DDI multi-classification

Since multi-classification DDI prediction provides a more valuable reference for medical practitioners, it is the focus of this paper. The performance comparison of our model with other state-of-the-art models is shown in Tables 6, 7.

**TABLE 6:** Comparison results of MSDF with other models in DDIMDL dataset.

Method	ACC	AUCPR	AUC	F1	Precision	Recall
DNN	0.880	0.913	0.996	0.722	0.805	0.703
DDIMDL	0.885	0.921	0.998	0.759	0.847	0.718
DeepDDI	0.837	0.890	0.996	0.685	0.728	0.661
AM-GCN	0.912	0.968	0.999	0.810	0.854	0.795
DM-DDI	0.908	0.964	0.999	0.852	0.879	0.839
MFDA	0.902	0.963	0.998	0.851	0.857	0.853
MSDF	**0.943**	**0.981**	**0.999**	**0.863**	**0.889**	**0.854**

The best results are shown in bold.

**TABLE 7:** Comparison results of MSDF with other models in DeepDDI dataset.

Method	ACC	AUCPR	AUC	F1	Precision	Recall
DNN	0.840	0.913	0.992	0.455	0.510	0.441
DDIMDL	0.939	0.968	0.999	0.903	0.918	0.900
DeepDDI	0.905	0.943	0.998	0.797	0.824	0.795
AM-GCN	0.924	0.979	0.999	0.880	0.904	0.875
DM-DDI	0.923	0.977	0.999	0.887	0.928	0.867
MFDA	0.940	0.986	0.999	0.908	0.928	0.900
MSDF	**0.972**	**0.995**	**0.999**	**0.946**	**0.957**	**0.941**

The best results are shown in bold.

Table 6 reveals the performance of various methods on the DDIMDL dataset. DNN, DDIMDL, and DeepDDI, which solely rely on the drug’s own features without incorporating topological features of the drug network, exhibit lower performance across all metrics compared to methods that utilize graph embedding techniques. Among the methods based on graph embedding techniques, AM-GCN performs poorly in general. This is because AM-GCN only uses the output of GCN as the embedding vector of the drug. However, GCN, in this context, can not effectively fuse topological and node features. In contrast, both DM-DDI and MFDA implement a cross-fertilization strategy, allowing for a more comprehensive integration of the drug’s topological and node features. Therefore, they lead to superior performance in most metrics. Although both DM-DDI and MFDA achieve good results, the method MSDF proposed in this paper performs even better. The superior performance of MSDF is due to the introduction of the multi-scale fusion module, which effectively combines information from different scales to enhance model results. This hypothesis is further supported by subsequent ablation experiments.

**FIGURE 3:**
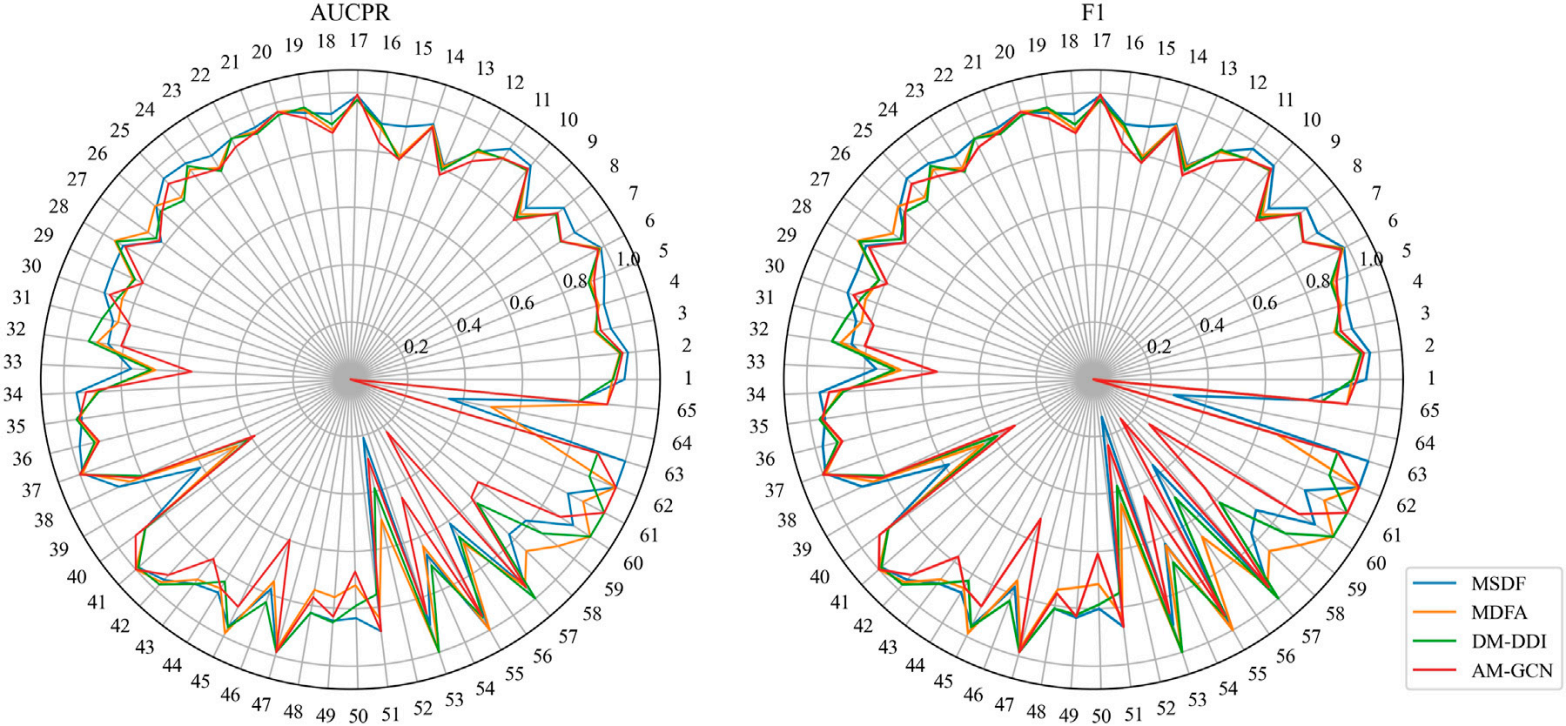
Comparison of AUPR and F1 predicted for each event in the DDIMDL dataset.

While MSDF generally outperforms the baseline method, the evaluation of multi-classification models requires not only observing the overall classification effectiveness, but also assessing the classification performance of individual categories. Thus, in our analysis of the DDIMDL dataset, we comprehensively evaluate the predictive performance across all categories using two metrics: AUCPR and F1 score. These results are visually depicted in the radar chart of Figure 3. In the radar plot of Figure 3, we compare the category-wise prediction performance of MSDF against three methods that demonstrate overall superior performance: AM-GCN, DM-DDI, and MFDA. Each spoke of the radar chart represents a different label category, arranged in descending order of label frequency occurrence. For example, the first spoke (number 1) represents the label with the highest frequency of occurrence, while the last spoke (number 65) corresponds to the label with the 65th frequency of occurrence.

**FIGURE 4:**
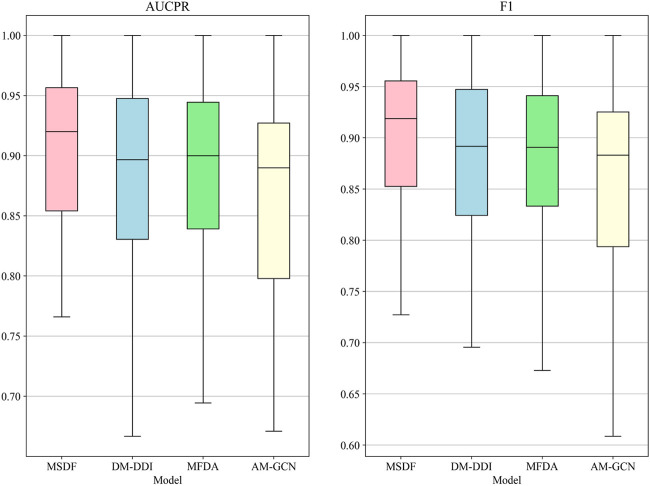
AUPR and F1 box plots for each tag in the DDIMDL dataset.

In the radar chart, it can be seen that MSDF achieves better or comparable results to other methods in predicting labels up to number 51. This is particularly notable for labels up to number 18, where MSDF outperforms comparison methods. For instance, in predicting label number 39, other methods show significantly lower results, falling below 50% in both AUPR and F1 scores. In contrast, MSDF’s predictions for this label are considerably higher, with AUPR at 60.9% and F1 at 58.5%. These observations suggest that it can be seen that MSDF excels in predicting labels that occur more frequently However, for labels numbered 51 and beyond MSDF does not fetch better performance. An analysis of label distribution reveals that labels after number 51 appear fewer than 10 times in the dataset, indicating extremely limited data. Labels after number 60 appear only 5 times. According to Kang et al. (Kang et al., 2022), GCN cannot achieve better performance in category-imbalanced data, which might explain MSDF’s lower performance in these rarer label predictions. To further analyze the predictive performance of the models, box plots of different models on AUCPR and F1 under 65 classifications are plotted in this paper, as shown in Figure 4. From the box plots, it can be clearly seen that MSDF achieves better performance from the statistical level.

**FIGURE 5:**
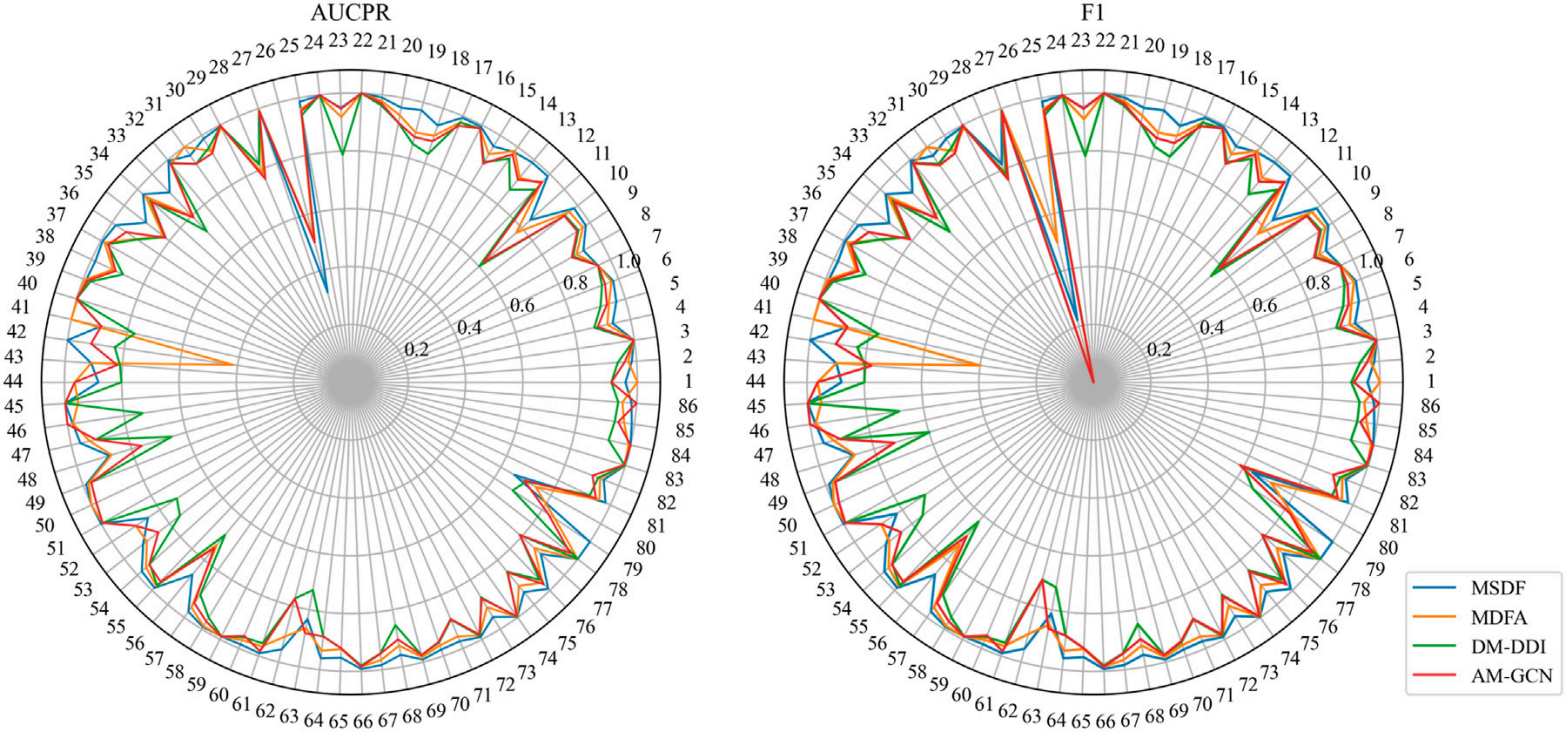
Comparison of AUPR and F1 predicted for each event in the DeepDDI dataset.

**FIGURE 6:**
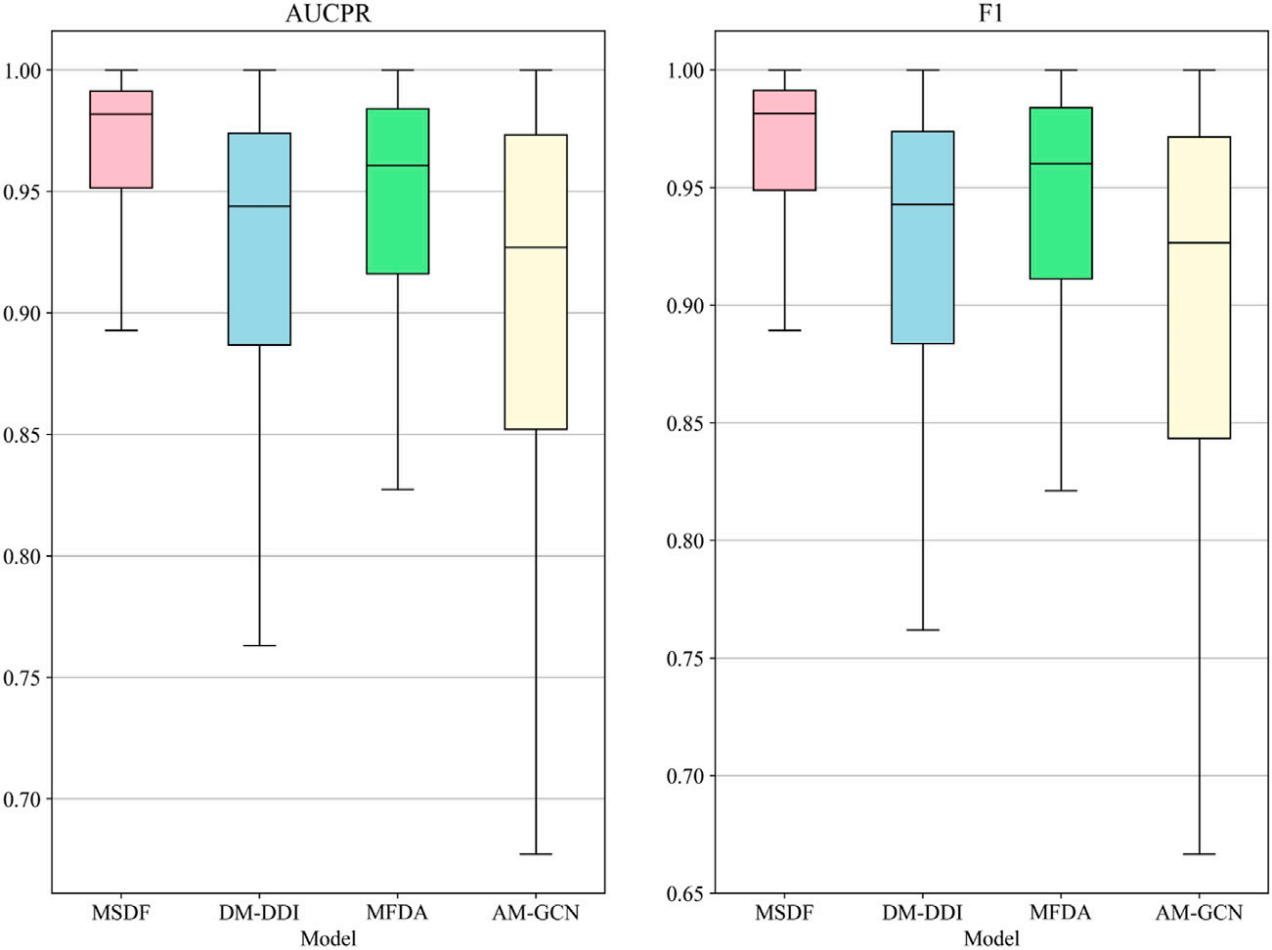
AUPR and F1 box plots for each tag in the DeepDDI dataset.

In the DeepDDI dataset, we obtain similar conclusions as in the DDIMDL dataset, i.e., our proposed method outperforms the other models in all metrics. However, by comparing the results of the two datasets, we can see that on the DeepDDI dataset, the metrics of each model except DNN are significantly improved compared to those on the DDIMDL dataset, and this paper speculates that this is because as the dataset grows larger, the information input to the model is richer and therefore more effective. Interestingly, the performance of AM-GCN on DeepDDI does not lag behind DM-DDI, unlike in the DDIMDL dataset. This paper suggests that this difference may be attributed to the sparser network structure in DDIMDL dataset, where the drug’s inherent features have a more significant impact on prediction outcomes. In this paper, we also evaluate the classification performance of individual categories on the DeepDDI dataset and the results are shown in Figures 5, 6. Unlike the DDIMDL dataset, the labels in the DeepDDI dataset are not ordered by the frequency of label occurrence. In Figure 5, it can be seen that MDSF achieves optimal results in most categories. From the box plots in Figure 6, it can be seen that MSDF achieves the best performance in the DeepDDI dataset at the statistical level as well.

### 4.7 Ablation experiments

To verify the effectiveness of the dual-view and multi-scale fusion modules in MSDF, this paper conducts ablation experiments. The results of the ablation experiments are shown in Table 8. Among them, “without multi-scale fusion” refers to experiments where only the dual-view approach is implemented, omitting the multi-scale fusion module, “without topological graph” indicates that only the feature graph and multi-scale fusion module are introduced in the experiments, Conversely, “without feature graph” means that the experiments were conducted with just the topological graph and the multi-scale fusion module. Based on the results in Table 8, the following conclusions can be drawn: (1) The removal of the multi-scale fusion module resulted in the largest decrease in model effectiveness, so the fusion of information from different scales is crucial. (2) The performance of the model decreases with the removal of either topological or feature view, which indicates that the introduction of two views for prediction is effective. (3) The model can be optimized by introducing two views with the multi-scale fusion module.

**TABLE 8:** Results of ablation experiments.

Dataset	Method	ACC	AUCPR	AUC	F1	Precision	Recall
DDIMDL	Without multi-scale fusion	0.889	0.954	0.998	0.748	0.804	0.724
	Without topological view	0.931	0.975	0.998	0.838	0.869	0.824
	Without feature view	0.928	0.977	0.999	0.853	0.887	0.837
	MSDF full model	**0.943**	**0.981**	**0.999**	**0.863**	**0.889**	**0.854**
DeepDDI	Without multi-scale fusion	0.954	0.990	0.999	0.913	0.940	0.901
	Without topological view	0.962	0.992	0.999	0.919	0.935	0.912
	Without feature view	0.966	0.994	0.999	0.926	0.939	0.921
	MSDF full model	**0.972**	**0.995**	**0.999**	**0.946**	**0.957**	**0.941**

The best results are shown in bold.

In this paper, we not only conduct experiments to validate the overall functionality of our model but also specifically investigate how information from different scales affects DDI prediction results. For this purpose we individually select and utilize information from each scale for DDI prediction. In our representation, “Scale-n” denotes the scale used, where “n” indicates the scale number. For instance, “Scale-1” refers to information from the first GCN layer, while “MSDF” represents the complete model that fuses information from all three layers for prediction. The outcomes of these focused experiments are detailed in Table 9. From Table 9, the following conclusions can be drawn: (1) Relatively effective predictions can be obtained from information at different scales; (2) The prediction effect is better for information at lower scales, indicating that as the number of GCN layers increases, the phenomenon of over-smoothing occurs, which reduces the prediction performance; (3) The best results can be obtained by fusing the three kinds of information, because the information is more comprehensive.

**TABLE 9:** Effect of different scale information on the results.

Dataset	Method	ACC	AUCPR	AUC	F1	Precision	Recall
DDIMDL	Scale-1	0.939	0.979	0.999	0.845	0.872	0.833
	Scale-2	0.923	0.972	0.999	0.812	0.855	0.797
	Scale-3	0.889	0.954	0.998	0.748	0.804	0.724
	MSDF full model	**0.943**	**0.981**	**0.999**	**0.863**	**0.889**	**0.854**
DeepDDI	Scale-1	0.971	0.995	0.999	0.936	0.952	0.927
	Scale-2	0.964	0.993	0.999	0.925	0.944	0.917
	Scale-3	0.954	0.990	0.999	0.913	0.940	0.901
	MSDF full model	**0.972**	**0.995**	**0.999**	**0.946**	**0.957**	**0.941**

The best results are shown in bold.

### 4.8 Parameter sensitivity analysis

**FIGURE 7:**
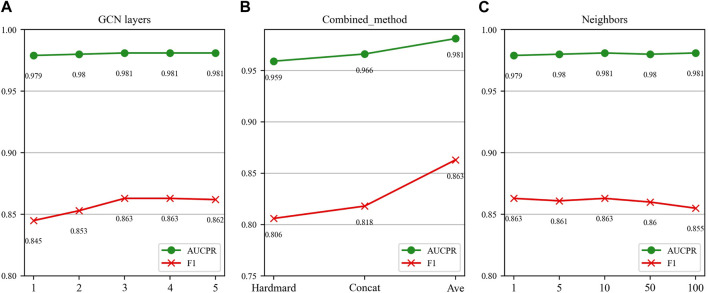
Parameter sensitivity analysis results in the DDIMDL dataset. **(A)** Effect of GCN layers. **(B)** Effect of drug combination method. **(C)** Effect of the number of neighbours.

**FIGURE 8:**
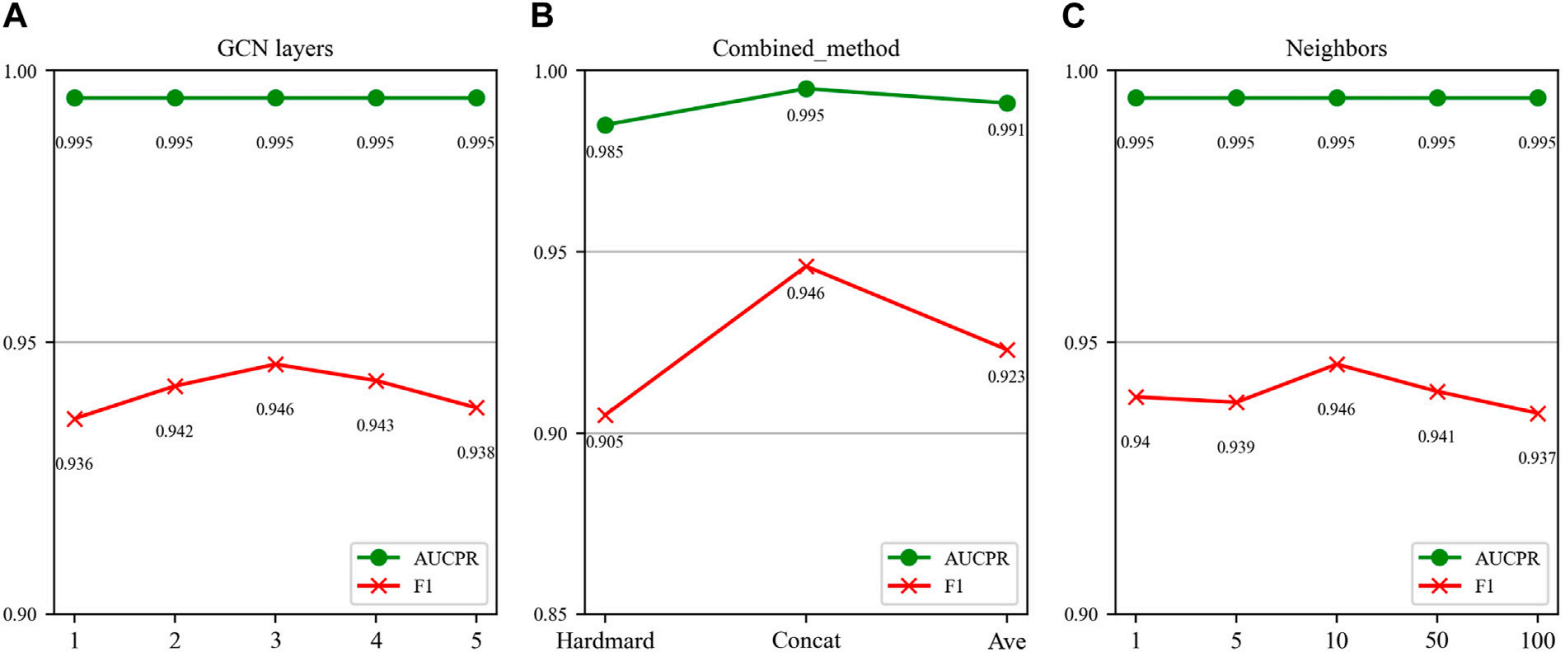
Parameter sensitivity analysis results in the DeepDDI dataset. **(A)** Effect of GCN layers. **(B)** Effect of drug combination method. **(C)** Effect of the number of neighbours.

In this section, we investigate the impact of three key parameters on our model’s performance: the number of GCN layers, the method of splicing drug pairs, and the number of neighbors in the feature graph. We employ AUPR and F1 scores as our primary evaluation metrics, as they are particularly relevant in multi-classification experiments. Figures 7, 8 illustrate the results of the study for these specific parameters in the DDIMDL and DeepDDI datasets, respectively.

In this paper, we first validate the effect of the number of GCN layers on the model. The experiment increases the number of GCN layers from 1 to 5, with the dimensions of each layer set as (256, 128, 64, 32, 16). After each increase in the layer count, we record the model’s performance. The results of the DDIMDL dataset are shown in Figure 7A, and results of the DeepDDI dataset are shown in Figure 8A. It can be seen that the model achieves the best performance when the number of GCN layers is 3. Beyond this, adding more layers does not further enhance the results, suggesting that three layers suffice for adequate information gathering.

Next, we assess how different methods of splicing drug pairs affect the model. This experiment reveals varying impacts on performance, as detailed in Figures 7B, 8B. In the DDIMDL dataset, the combined “averaging” method proved to be the most effective, while in the DeepDDI dataset, the combined “concat” method proved to be the most effective.

Lastly, we explore the influence of the number of neighbors in feature graph construction. For this, this paper conduct experiments with varying number of neighbors set (1, 5, 10, 50, 100). As can be seen in Figures 7C, 8C, when the number of neighbors is less than 10, the parameter has a negligible impact on the results. However, when the number of neighbors reaches 100, the model’s performance significantly declines. This decrease indicates that accumulating too many neighbors with low similarity introduces noise. Consequently, in our feature graph construction, we select the 10 nodes with the highest similarity to the target node as its neighbors.

### 4.9 Case study

To demonstrate the practical utility of the model, we trained it using known DDIs from the DeepDDI dataset and the DDIMDL dataset, respectively. After training, the model was used to predict the likelihood of DDIs for drug pairs not present in the dataset. We then selected the top 10 drug pairs with the highest probability of predicting DDI in each of the two datasets and searched for evidence of these interactions in the Drugbank database. The results of this case study are presented in Table 10 the method proposed in this paper achieves a good accuracy rate with 14 out of 20 predictions are validated in the Drugbank dataset. For example, an interaction between the drug Digoxin and the drug Roflumilast is described as “Roflumilast may decrease the excretion rate of Digoxin which could result in a higher serum level.” Therefore, the model proposed in this paper is of practical utility. However, among the twenty DDIs with the highest predictive scores, six pairs: “Estrone-Drospirenone,” “Olopatadine-Quinine,” “Benzphetamine-Cholecalciferol,” “Acetylsalicylicacid-Bortezomib,” “Halothane-Azithromycin,” and “Fingolimod-Amrubicin” failed to find the corresponding evidence in drugbank. In this paper, we argue that this could be the result of an error in the model predictions, but it could also be a DDI that is currently unrecognized by people. Hence, extra attention is needed for these six pairs of DDIs.

**TABLE 10:** The 20 DDIs with the highest predicted probabilities.

Dataset	Rank	DrugA	DrugB	Source	Description
DeepDDI	1	Pramipexole	Desvenlafaxine	Drugbank	Desvenlafaxine may increase the sedative activities of Pramipexole
	2	Warfarin	Probenecid	Drugbank	The metabolism of Warfarin can be increased when combined with Probenecid
	3	Digoxin	Roflumilast	Drugbank	Roflumilast may decrease the excretion rate of Digoxin which could result in a higher serum level
	4	Phenytoin	Nicergoline	Drugbank	The metabolism of Nicergoline can be decreased when combined with Phenytoin
	5	Estrone	Drospirenone	N.A.	N.A.
	6	Olopatadine	Quinine	N.A.	N.A.
	7	Triptorelin	Ivabradine	Drugbank	The risk or severity of QTc prolongation can be increased when Triptorelin is combined with Ivabradine
	8	Benzphetamine	Cholecalciferol	N.A.	N.A.
	9	Bosentan	Thioridazine	Drugbank	Thioridazine may decrease the antihypertensive activities of Bosentan
	10	Methysergide	Carbamazepine	Drugbank	The metabolism of Methysergide can be increased when combined with Carbamazepine
DDIMDL	1	Dronabinol	Desvenlafaxine	Drugbank	Dronabinol may increase the central nervous system depressant (CNS depressant) activities of Desvenlafaxine
	2	Dronabinol	Escitalopram	Drugbank	Dronabinol may increase the central nervous system depressant (CNS depressant) activities of Escitalopram
	3	Mifepristone	Ceritinib	Drugbank	The serum concentration of Ceritinib can be decreased when it is combined with Mifepristone
	4	Acetylsalicylicacid	Bortezomib	N.A.	N.A.
	5	Bortezomib	Etodolac	Drugbank	The metabolism of Etodolac can be decreased when combined with Bortezomib
	6	Citalopram	Losartan	Drugbank	Losartan may increase the QTc-prolonging activities of Citalopram
	7	Chlorpromazine	Doxorubicin	Drugbank	The metabolism of Doxorubicin can be increased when combined with Chlorpromazine
	8	Halothane	Azithromycin	N.A.	N.A.
	9	Fingolimod	Amrubicin	N.A.	N.A.
	10	Fluvoxamine	Amantadine	Drugbank	The risk or severity of serotonin syndrome can be increased when Fluvoxamine is combined with Amantadine

## 5 Conclusion

In this paper, we propose a novel DDI prediction model, MSDF, which takes topology and feature graphs as inputs and fully integrates the information from the outputs of the GCN layers through an attention mechanism to obtain multi-scale drug embedding vectors. The advantages of this method are that the limitation of utilising only one view is alleviated by introducing a dual view, and the information of drug feature vectors containing multi-scale information is more comprehensive. However, the method proposed in this paper still has a shortcoming: there is no obvious advantage in the prediction performance in the extremely rare category. However, on the whole, the method proposed in this paper achieves better results than the baseline method on datasets of different scales, reflecting the superiority of the MSDF model for DDI prediction. Meanwhile, there is still room for improvement in the model of this paper, firstly, the binary classification and multi-classification tasks are completed in the experiments of this paper, but the multi-label problem is not investigated. Secondly, this paper uses GCN to aggregate the information of drug nodes, but GCN suffers from the problem of poor performance in category unbalanced data and excessive smoothing, so the introduction of a novel graph neural network technique to aggregate the information of drug neighboring nodes is also an idea to improve the effectiveness of the model.

## Data Availability

Publicly available datasets were analyzed in this study. This data can be found here: https://github.com/YifanDengWHU/DDIMDL https://bitbucket.org/kaistsystemsbiology/deepddi/src/master/.
